# Longitudinal associations between physical activity and negative emotions among college students: the chain mediating roles of exercise self-efficacy and psychological resilience

**DOI:** 10.3389/fpsyg.2026.1881725

**Published:** 2026-08-04

**Authors:** Yibo Yue, Dong Zhu, Quan Zeng, Chang Hu, Bo Xu

**Affiliations:** 1 School of Physical Education, Shaoyang University, Shaoyang, China; 2 Hunan University of Medicine, Huaihua, Hunan, China; 3 Department of Rehabilitation Medicine, Hunan University of Medicine General Hospital, Huaihua, Hunan, China; 4 Physical Education College, Jiangxi Normal University, Nanchang, China

**Keywords:** chain mediation, college students, exercise self-efficacy, longitudinal study, negative emotions, physical activity, psychological resilience

## Abstract

**Objective:**

This study examined the longitudinal association between physical activity and negative emotions among Chinese college students and tested the chain mediating roles of exercise self-efficacy and psychological resilience.

**Methods:**

A three-wave longitudinal study was conducted over 1 year among students from two universities in Hunan and Jiangxi Provinces, China. The final analytic sample included 877 college students. Physical activity and baseline negative emotions were assessed at Time 1, exercise self-efficacy at Time 2, and psychological resilience and negative emotions at Time 3. Descriptive statistics, Pearson correlations, and chain mediation analyses were conducted using SPSS 26.0 and the PROCESS macro. Gender, grade, and baseline negative emotions were controlled.

**Results:**

After controlling for covariates, T1 physical activity was negatively associated with T3 negative emotions, *β* = −0.192, *p* < 0.001. T1 physical activity was positively associated with T2 exercise self-efficacy, *β* = 0.355, *p* < 0.001, and T3 psychological resilience, *β* = 0.210, *p* < 0.001. T2 exercise self-efficacy was positively associated with T3 psychological resilience, *β* = 0.225, *p* < 0.001, and negatively associated with T3 negative emotions, *β* = −0.173, *p* < 0.001. T3 psychological resilience was also negatively associated with T3 negative emotions, *β* = −0.105, *p* < 0.01. The total indirect association was significant, effect = −0.092, 95% CI [−0.121, −0.065], as was the sequential indirect association involving T2 exercise self-efficacy and T3 psychological resilience, effect = −0.008, 95% CI [−0.015, −0.003].

**Conclusion:**

Physical activity was associated with lower negative emotions among college students, partly through higher exercise self-efficacy and psychological resilience. These findings provide longitudinal evidence for university-based mental health promotion through physical activity.

## 1 Introduction

In recent years, negative emotions among college students have become an increasingly important concern in public health, educational psychology, and university mental health research (Huang et al., 2025a; Zhang W. et al., 2025; Zhang et al., 2026b). The college years represent a critical developmental period in which individuals transition from late adolescence to early adulthood. During this stage, students are required to navigate multiple developmental and contextual challenges, including academic competition, adjustment to their major, interpersonal relationships, career planning, and uncertainty about the future, while simultaneously developing a clearer sense of identity and greater independence in daily life (Yang et al., 2026; Hu et al., 2026a; Yuan et al., 2026). These accumulated demands may increase vulnerability to negative emotional experiences, particularly symptoms of depression, anxiety, and stress (Hu et al., 2026d). Negative emotions generally refer to adverse affective states that emerge in response to stress, setbacks, or perceived threats and are commonly manifested as depressive, anxious, and stress-related symptoms (Iannattone et al., 2024). Evidence from systematic reviews and meta-analyses indicates that such symptoms are highly prevalent among college students, with pooled prevalence estimates of approximately 29% for anxiety, 39% for depression, and 23% for stress (Li et al., 2022; Paiva et al., 2025; Ahmed et al., 2023). These findings suggest that negative emotions constitute a widespread and pressing mental health issue in this population. Similar concerns have been reported among Chinese college students. A previous meta-analysis estimated the overall prevalence of depressive symptoms in this group to be approximately 28.4%, and more recent evidence further indicates that depression remains a substantial and persistent public health burden among college students in China (Wang et al., 2023).

Persistent negative emotions may substantially undermine college students’ psychological well-being and are often associated with poorer academic engagement, impaired sleep quality, interpersonal difficulties, reduced social adjustment, and lower life satisfaction, thereby increasing the risk of more serious mental health problems (Yue et al., 2025b; Hu et al., 2026b; Huang D. et al., 2024). Importantly, negative emotions among college students are not only individual psychological experiences but are also closely related to the quality of higher education, the effectiveness of campus support systems, and the long-term development of young adults (Wang et al., 2020). Although mental health education, counseling services, and crisis intervention systems in universities have continued to improve, many existing services remain primarily oriented toward problem identification and *post hoc* intervention. For students who do not meet clinical diagnostic thresholds but already experience notable emotional distress, more proactive, universal, and sustainable mental health promotion strategies are still needed. Therefore, identifying protective factors that can be modified and strengthened in daily life is of considerable theoretical and practical significance for promoting college students’ mental health and improving university-based prevention and intervention systems.

Among the modifiable factors that may support mental health, physical activity has been widely regarded as an important behavioral resource associated with lower levels of negative emotions among college students (Huang et al., 2025b; Yuan et al., 2025). Physical activity generally refers to any bodily movement produced by skeletal muscles that results in energy expenditure (Hu et al., 2025a). It includes not only planned, structured, and repetitive exercise but also movement embedded in daily life, transportation, leisure activities, and campus routines (Yue et al., 2025a; Huang et al., 2026). Compared with specialized psychological interventions alone, physical activity is relatively low-cost, scalable, sustainable, and easily integrated into students’ everyday lives, making it particularly valuable for mental health promotion in university settings (Chen et al., 2026; Liu et al., 2026; Zhang et al., 2026a).

However, physical inactivity has become increasingly common among college students, partly due to prolonged sedentary study, online entertainment, extensive mobile internet use, and growing academic pressure (Wan et al., 2025; Kwok et al., 2021; Teuber et al., 2024). Insufficient physical activity may impair physical functioning and sleep quality, reduce opportunities for positive emotional experiences and in-person social interaction, and weaken students’ sense of control over their own state and daily routines (Belluardo et al., 2025; Ghrouz et al., 2019). These changes may, in turn, increase the risk of depression, anxiety, and stress. Consistent with this view, previous studies have shown that higher levels of physical activity are closely associated with lower levels of depression, anxiety, and stress, and that students who engage in regular physical activity tend to report better emotional states and psychological adjustment (Zhang et al., 2022; Pascoe et al., 2020; Zhang L. et al., 2025). Physical activity may benefit mental health by improving physical health, fostering positive emotions, enhancing self-esteem, strengthening stress-coping capacity, and increasing opportunities for social interaction (Zou et al., 2022; Chen et al., 2025a; Chen et al., 2025b).

Nevertheless, much of the existing evidence relies on cross-sectional designs or short-term follow-up studies (Singh et al., 2023; Liu et al., 2024). Although these studies have established associations between physical activity and negative emotions, they cannot fully clarify the temporal ordering of these variables or explain the psychological mechanisms through which physical activity may be linked to later emotional outcomes. This limitation is especially important for college students, who are undergoing a major developmental transition and whose emotional states are often highly variable. In the present study, depression, anxiety, and stress were combined into a broader construct of negative emotions because these symptoms are theoretically related, frequently co-occur, and are commonly regarded as overlapping manifestations of general psychological distress. This approach allows the study to capture a more comprehensive profile of adverse emotional experiences among college students. However, depression, anxiety, and stress are also distinct psychological constructs with different symptom characteristics and may not be associated with physical activity in the same way. For example, physical activity may be more closely related to depressive symptoms through behavioral activation and positive affect, whereas its association with anxiety or stress may involve perceived control, physiological regulation, or stress-coping processes. Therefore, whether physical activity is longitudinally associated with changes in overall negative emotions, and whether this association operates through stable psychological resources, remains to be further examined.

Exercise self-efficacy may be an important psychological mechanism linking physical activity to negative emotions. Exercise self-efficacy refers to an individual’s confidence in their ability to initiate, maintain, and overcome barriers to physical activity (Hu et al., 2025b). It represents a positive self-belief within the exercise context. According to social cognitive theory, individuals’ behavioral experiences shape their efficacy beliefs, which in turn influence motivation, persistence, and emotional responses (Bandura, 2001). Through participation in physical activity, college students may gradually develop stronger confidence in their exercise capacity and behavioral control by improving motor skills, achieving exercise goals, enhancing physical fitness, and receiving positive feedback (Meng et al., 2022; Lin et al., 2025).

Higher exercise self-efficacy may not only help students maintain regular physical activity, but also strengthen their perceived control and active coping tendencies when facing stressful situations (Sheng et al., 2025; Qin et al., 2025). When students believe that they can take action to improve their situation, overcome difficulties, and achieve goals, they may be more likely to adopt adaptive emotion regulation strategies in response to academic pressure, interpersonal conflict, and daily life challenges (Parada and Verlhiac, 2021; Meyer and Stutts, 2024). As a result, they may experience lower levels of depression, anxiety, and stress. Thus, physical activity may reduce negative emotions partly by enhancing college students’ exercise self-efficacy.

In addition to exercise self-efficacy, psychological resilience may also serve as an important psychological mechanism linking physical activity to negative emotions (Zhang S., 2025). Psychological resilience refers to the capacity to maintain adaptive functioning, recover effectively, and continue to develop when facing stress, adversity, or challenges (Troy et al., 2023; Fletcher and Sarkar, 2013). It is an important protective resource for college students’ mental health. Physical activity is often characterized by challenge, goal orientation, and persistence (Huang K. et al., 2024). During exercise, individuals must repeatedly cope with fatigue, difficulty, setbacks, and demands for self-regulation, all of which may provide a real-life context for the development of resilience (Çakir et al., 2025).

Regular physical activity may help college students accumulate successful experiences in overcoming challenges, strengthen persistence, frustration tolerance, and stress endurance, and foster more adaptive emotion regulation strategies (Sun et al., 2026; Xiang et al., 2026; Gou et al., 2026). Previous studies have also shown that individuals with higher levels of psychological resilience are better able to maintain positive cognition, regulate emotions flexibly, and mobilize internal and external resources when facing stressful events (Mak et al., 2011; Ong et al., 2006). Consequently, they tend to report lower levels of depression, anxiety, and stress (Polizzi and Lynn, 2021). Taken together, physical activity may further reduce negative emotions among college students by enhancing psychological resilience.

Exercise self-efficacy and psychological resilience are not entirely independent psychological resources; rather, they may operate in a sequential manner. Exercise self-efficacy primarily reflects individuals’ positive beliefs about their ability and behavioral control within the exercise context, whereas psychological resilience reflects their capacity to adapt and recover in broader stressful situations (Neupert et al., 2009; Jiang et al., 2025). From the perspective of psychological resource development, positive self-beliefs formed through physical activity may gradually transfer and expand into more general resources for psychological adaptation (Xing and Ge, 2025; Leng et al., 2025).

Specifically, higher exercise self-efficacy may strengthen college students’ confidence in their own capacity for action, making them more likely to adopt active coping strategies rather than avoidance or withdrawal when facing difficulties (Li et al., 2024; Zhang R., 2025). At the same time, exercise self-efficacy may further promote psychological resilience by reinforcing goal persistence, behavioral control, and self-regulation (Halper and Vancouver, 2015). In other words, when students repeatedly experience that they can persist, achieve goals, and improve their own condition through physical activity, these positive efficacy beliefs may gradually be transformed into resilience and adaptability in response to academic stress, interpersonal difficulties, and daily life challenges (Belcher et al., 2020). Conversely, students with lower exercise self-efficacy may be more likely to doubt their own abilities and show weaker active coping and emotion regulation in stressful situations, which may hinder the development of psychological resilience (Michinov et al., 2023; Cattelino et al., 2021). Thus, physical activity may first enhance college students’ exercise self-efficacy, which may then strengthen psychological resilience and ultimately reduce negative emotions.

Taken together, building on previous research, the present study adopts a three-wave longitudinal design to further examine the longitudinal association between physical activity and negative emotions among college students. It also tests the independent mediating roles of exercise self-efficacy and psychological resilience, as well as their chain mediating effect. Compared with previous studies that have primarily relied on cross-sectional data or single-mediator models, this study is better positioned to clarify the temporal ordering among physical activity, exercise self-efficacy, psychological resilience, and negative emotions. By addressing these issues, the present study may deepen our understanding of the mechanisms through which physical activity promotes college students’ mental health and provide theoretical and empirical support for university-based mental health promotion and interventions targeting negative emotions.

### 1.1 The present study and hypotheses

**Figure 1:**
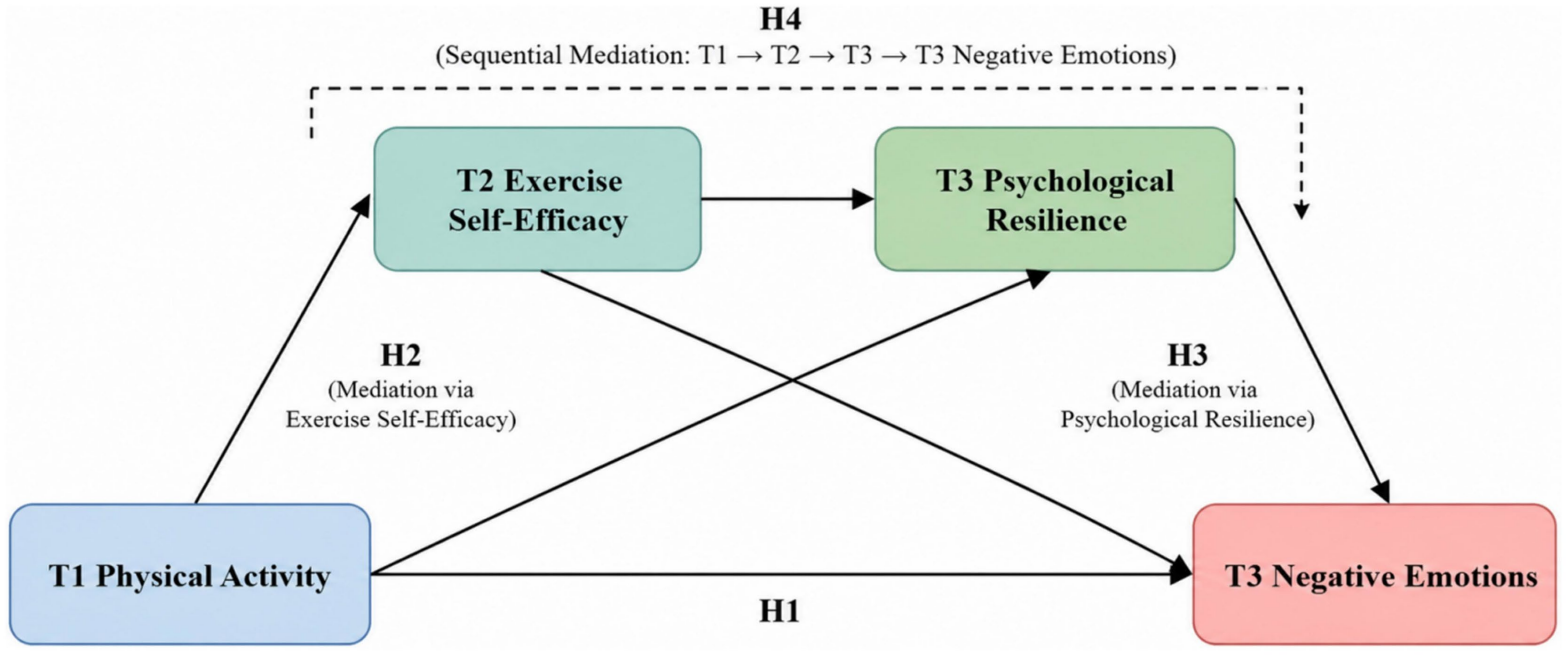
Hypothesized longitudinal chain mediation model of the association between physical activity and negative emotions among college students.

Based on the theoretical rationale outlined above, we developed a hypothesized longitudinal chain mediation model linking T1 physical activity, T2 exercise self-efficacy, T3 psychological resilience, and T3 negative emotions, as shown in Figure 1. Accordingly, the following hypotheses were proposed:

*Hypothesis 1*: T1 physical activity would be negatively associated with T3 negative emotions among college students.

*Hypothesis 2*: T2 exercise self-efficacy would mediate the longitudinal association between T1 physical activity and T3 negative emotions among college students.

*Hypothesis 3*: T3 psychological resilience would mediate the longitudinal association between T1 physical activity and T3 negative emotions among college students.

*Hypothesis 4*: T2 exercise self-efficacy and T3 psychological resilience would jointly serve as chain mediators in the longitudinal association between T1 physical activity and T3 negative emotions among college students.

## 2 Methods

### 2.1 Participants and procedure

This study was a three-wave longitudinal cohort study designed to examine the longitudinal association between physical activity and negative emotions among Chinese college students, as well as the chain mediating roles of exercise self-efficacy and psychological resilience. Participants were recruited from two universities located in Hunan Province and Jiangxi Province, China. A convenience sampling method was used because of the feasibility of participant recruitment and longitudinal follow-up. Although the sample was not randomly selected, recruiting students from universities in two different provinces helped improve the heterogeneity of the sample to some extent.

Data were collected at three time points over 1 year. The baseline survey was conducted offline in classroom settings from April 12 to April 27, 2025. The second and third waves were conducted online approximately 6 months and 12 months after baseline, respectively. The offline baseline survey enabled the research team to explain the study purpose, obtain informed consent, and establish a stable participant identification system. The subsequent online surveys were used to facilitate follow-up, reduce participant burden, and improve response efficiency after students returned to their regular classes, campuses, or daily routines.

At baseline, a total of 1,008 college students participated in the survey. After excluding invalid questionnaires, including responses with substantial missing data, implausibly short response times, failed attention-check items, or inconsistent mixed identification codes, 926 valid participants were retained at T1. All eligible baseline participants were invited to participate in the follow-up surveys. Ultimately, 910 participants completed the T2 assessment, and 903 participants completed the T3 assessment. Participants who completed all three waves and whose responses could be successfully matched across waves were included in the final longitudinal analyses. The final analytic sample consisted of 877 college students.

**Figure 2:**
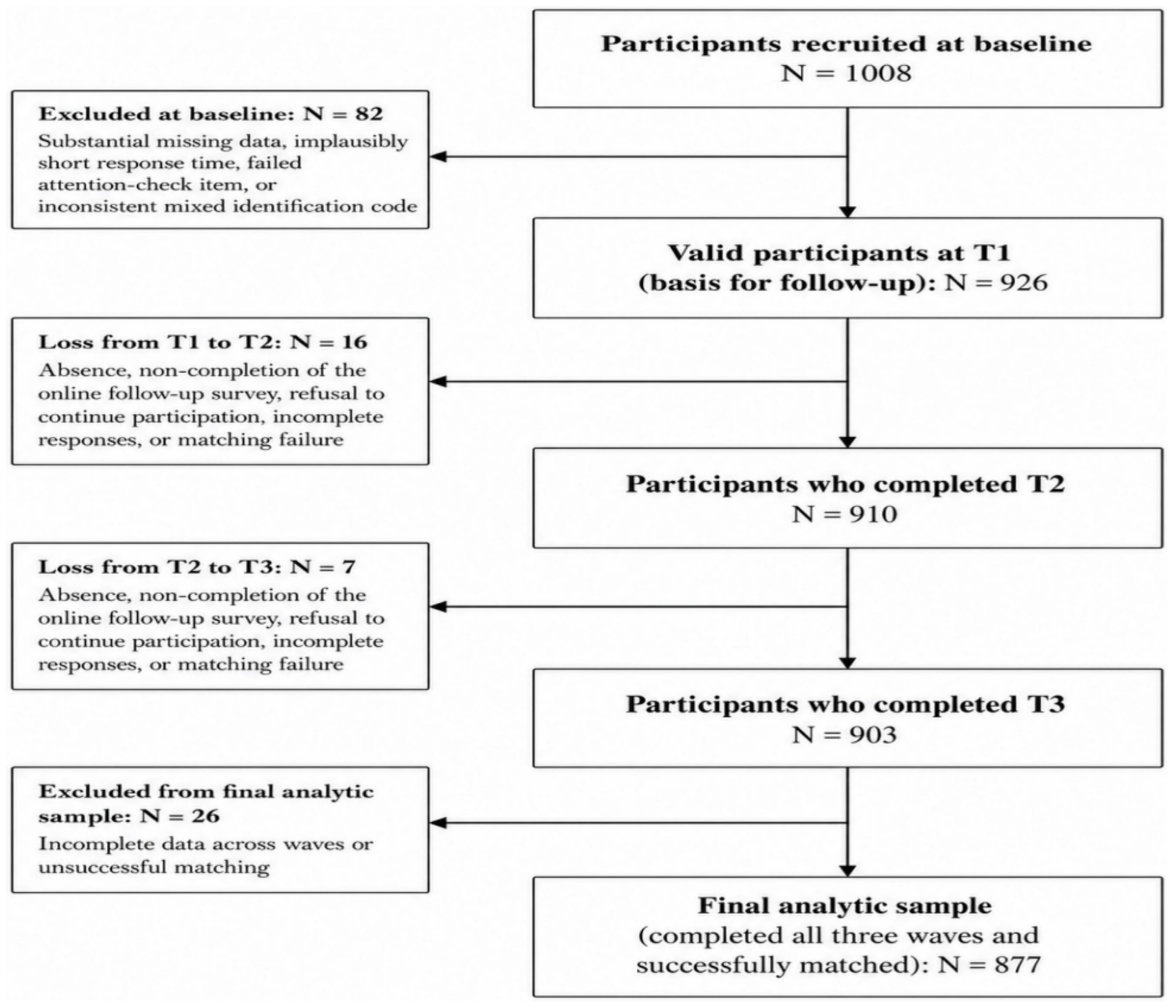
Flowchart of participant recruitment, follow-up, and final sample inclusion in the three-wave longitudinal study.

Among the final sample, 339 participants were male, accounting for 38.7%, and 538 were female, accounting for 61.3%. Regarding grade level, 422 participants were freshmen, accounting for 48.1%; 325 were sophomores, accounting for 37.1%; and 130 were juniors, accounting for 14.8%. In terms of only-child status, 417 participants were only children, accounting for 47.5%, whereas 460 were not only children, accounting for 52.5%. Regarding residence, 393 participants came from rural areas, accounting for 44.8%, and 484 came from urban areas, accounting for 55.2%. Participants’ ages ranged from 18 to 23 years, with a mean age of 19.68 years and a standard deviation of 1.21. The recruitment and retention of participants are shown in Figure 2.

Variables measured at each wave were arranged according to the hypothesized longitudinal chain mediation model. At T1, participants reported demographic information, physical activity, and baseline negative emotions. At T2, exercise self-efficacy was assessed. At T3, psychological resilience and negative emotions were assessed. Negative emotions included symptoms of depression, anxiety, and stress. The assessment of negative emotions at both T1 and T3 allowed the study to account for baseline levels of negative emotions when examining whether T1 physical activity predicted subsequent negative emotions at T3. This measurement arrangement also enabled the study to test whether the longitudinal association between T1 physical activity and T3 negative emotions was mediated by T2 exercise self-efficacy and T3 psychological resilience.

To match responses across the three waves while protecting participants’ privacy, each participant was asked to generate a mixed identification code according to a standardized rule. Specifically, the code consisted of the last four digits of the participant’s mobile phone number combined with the last two digits of their student ID number. This mixed code was used only for longitudinal data matching and was not included as directly identifiable personal information in the analytic dataset. For the online follow-up surveys, participants completed the questionnaires through a secure online survey platform and entered the same mixed identification code. The research team checked the consistency of mixed codes, demographic information, and response patterns to ensure accurate matching across waves.

The main reasons for sample attrition included absence on the day of assessment, failure to complete the online follow-up survey, refusal to continue participation, incomplete responses, and unsuccessful matching due to missing or inconsistent mixed identification codes. To examine whether attrition may have influenced the findings, baseline negative emotion scores were compared between participants who completed all three waves and those who dropped out after the first wave. The results showed no significant difference between the retained and attrition samples in baseline negative emotions, suggesting that attrition was unlikely to substantially bias the main outcome variable.

Before data collection, participants were informed that the study used a longitudinal design and that two additional follow-up assessments would be conducted over the following year. Participation was entirely voluntary, and participants could withdraw from the study at any time without penalty. To ensure data quality, all research staff received standardized training before baseline data collection. During the offline baseline survey, trained researchers provided unified instructions and answered procedural questions without influencing participants’ responses. During the online follow-up surveys, the questionnaire system recorded response time, and attention-check items were included to identify careless responses. Questionnaires with substantial missing data, implausibly short completion times, failed attention checks, or inconsistent mixed identification codes were excluded. All participants provided informed consent before completing the survey. The study procedures were approved by the Ethics Committee of Jiangxi Normal University, with the approval number IRB-JXNU-PEC-20240105.

### 2.2 Measures

#### 2.2.1 Negative emotions

Negative emotions were assessed at T1 and T3 using the 21-item Depression Anxiety Stress Scale (DASS-21), developed by Lovibond (Lovibond and Lovibond, 1995). The DASS-21 is a widely used self-report instrument for assessing symptoms of depression, anxiety, and stress, and it has demonstrated good reliability and validity in Chinese student populations (Huang et al., 2025a; Hu et al., 2026b). The scale consists of three subscales: depression, anxiety, and stress, with seven items in each subscale. Participants rated how much each statement applied to them during the past week on a 4-point Likert scale ranging from 0 (did not apply to me at all) to 3 (applied to me very much or most of the time). Example items include “I found it difficult to relax” and “I felt down-hearted and blue.” In this study, item scores were summed to obtain a total negative emotion score, with higher scores indicating higher levels of negative emotions. The DASS-21 demonstrated good internal consistency in the present sample, with Cronbach’s *α* values of 0.818 at T1 and 0.808 at T3.

#### 2.2.2 Physical activity

Physical activity was assessed at T1 using the Physical Activity Rating Scale-3 (PARS-3), revised by Liang (Liang, 1994). The PARS-3 is a commonly used self-report instrument for assessing physical activity participation among Chinese students (Hu et al., 2026d; Hu et al., 2026c). The scale evaluates physical activity from three dimensions: exercise intensity, duration, and frequency. Each dimension is rated on a 5-point scale. The total physical activity score is calculated using the formula: intensity × (duration − 1) × frequency, with possible scores ranging from 0 to 100. Higher scores indicate higher levels of physical activity participation. According to established cutoff criteria, physical activity levels can be classified as low (19 or below), moderate (20 to 42), and high (43 or above). In the present study, the PARS-3 demonstrated acceptable internal consistency, with a Cronbach’s *α* of 0.780 at T1.

#### 2.2.3 Exercise self-efficacy

Exercise self-efficacy was assessed at T2 using the Exercise Self-Efficacy Scale. The scale was originally developed by Jerusalem and Schwarzer (2013) and later adapted by Hu et al. (2025b) for use in Chinese populations, showing satisfactory reliability and validity. This scale consists of 10 items and measures a unidimensional construct of confidence in one’s ability to participate in, persist in, and overcome difficulties during physical exercise. Participants rated each item on a 4-point Likert scale ranging from 1 (completely disagree) to 4 (completely agree). Example items include “I believe I can persist in achieving my exercise goals” and “I am convinced that I can handle unexpected challenges during physical exercise.” Item scores were summed, with higher scores indicating higher levels of exercise self-efficacy. The scale demonstrated good internal consistency in the present sample, with a Cronbach’s *α* of 0.824 at T2.

#### 2.2.4 Psychological resilience

Psychological resilience was assessed at T3 using the 10-item Connor-Davidson Resilience Scale, CD-RISC-10. The 10-item version was later developed by Campbell-Sills et al. (Campbell-Sills and Stein, 2007). The Chinese version has been translated and revised by Wang and has been widely used to assess psychological resilience in Chinese populations (Wollny and Jacobs, 2023). The CD-RISC-10 is a unidimensional self-report scale that measures individuals’ capacity to adapt to change, cope with stress, and recover from adversity. Participants rated each item on a 5-point Likert scale ranging from 0 (never) to 4 (always). Example items include “I am able to adapt when changes occur” and “I can stay focused and think clearly under pressure.” Item scores were summed to obtain a total score, with higher scores indicating higher levels of psychological resilience. The scale demonstrated good internal consistency in the present sample, with a Cronbach’s *α* of 0.824 at T3.

### 2.3 Data analysis

First, SPSS 26.0 was used to conduct descriptive statistics, reliability analyses, and Pearson’s correlation analyses for the main study variables. Descriptive statistics included means, standard deviations, frequencies, and percentages where appropriate. Cronbach’s α coefficients were calculated to evaluate the internal consistency of each scale. Pearson’s correlation analyses were conducted to examine the bivariate associations among T1 physical activity, T2 exercise self-efficacy, T3 psychological resilience, T1 negative emotions, and T3 negative emotions.

Second, gender, grade level, and baseline negative emotions were included as control variables in the analyses. Previous studies have suggested that gender and grade level may be associated with college students’ physical activity participation and mental health outcomes (Gao et al., 2019; Cen et al., 2025). In addition, baseline levels of negative emotions may influence subsequent negative emotions. Therefore, gender, grade level, and T1 negative emotions were controlled for in the longitudinal chain mediation model. Gender was dummy-coded as 0 = male and 1 = female. Grade level was dummy-coded, with freshmen used as the reference group, and two dummy variables created for sophomores and juniors.

Third, before conducting the mediation analysis, the assumptions of the data were examined. The skewness and kurtosis coefficients of the main variables were checked to evaluate approximate normality. Variance Inflation Factor values were also calculated to assess multicollinearity. The results showed that the skewness and kurtosis values of the variables were within an acceptable range, and all VIF values were below the commonly recommended threshold of 5, indicating that there was no serious multicollinearity (Marcoulides and Raykov, 2019). These results suggested that the data were suitable for subsequent regression-based mediation analyses.

Finally, the longitudinal chain mediation model was tested using the PROCESS macro in SPSS (Hayes et al., 2017). Specifically, Model 6 was used to examine whether T2 exercise self-efficacy and T3 psychological resilience sequentially mediated the association between T1 physical activity and T3 negative emotions. In this model, T1 physical activity was entered as the independent variable, T2 exercise self-efficacy as the first mediator, T3 psychological resilience as the second mediator, and T3 negative emotions as the dependent variable. Gender, grade level, and T1 negative emotions were entered as covariates. The bias-corrected bootstrap method with 5,000 resamples was used to test the significance of indirect effects. An indirect effect was considered statistically significant if the 95% bootstrap confidence interval did not include zero (Preacher and Hayes, 2004).

## 3 Results

### 3.1 Descriptive statistics and correlations

Table 1 presents the descriptive statistics and Pearson’s correlations among the main study variables. Before the mediation analysis, the distributional characteristics of the variables were examined, and the skewness and kurtosis values were within acceptable ranges. Scatterplot diagnostics also suggested approximately linear relationships among the variables, with no obvious influential outliers; therefore, Pearson’s correlation coefficients were used. The results showed that T1 physical activity was positively correlated with T2 exercise self-efficacy and T3 psychological resilience, and negatively correlated with T3 negative emotions. T2 exercise self-efficacy was positively correlated with T3 psychological resilience and negatively correlated with T3 negative emotions. T3 psychological resilience was also negatively correlated with T3 negative emotions. Overall, these findings provide preliminary support for the proposed longitudinal chain mediation model.

**Table 1:** Descriptive statistics and Pearson correlations among the main study variables.

Variables	M	SD	1	2	3	4
1. T1 physical activity	34.606	31.369	1			
2. T2 exercise self-efficacy	27.206	5.579	0.415**	1		
3. T3 psychological resilience	22.303	6.655	0.333**	0.356**	1	
4. T3 negative emotions	18.917	9.217	−0.382**	−0.409**	−0.319**	1

**p < 0.01.

### 3.2 Chain mediation effect analysis

**Figure 3:**
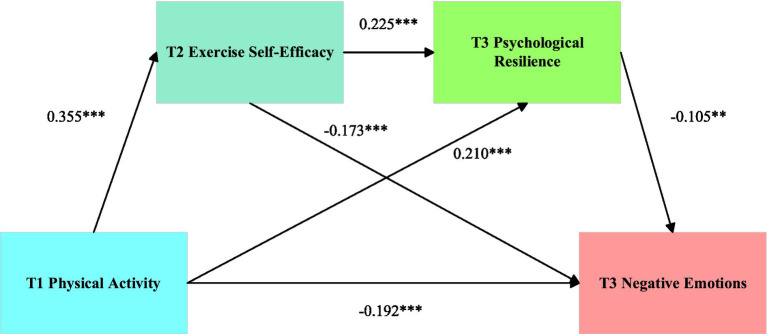
Tested longitudinal chain mediation model of the association between T1 physical activity and T3 negative emotions through T2 exercise self-efficacy and T3 psychological resilience. Standardized path coefficients are shown. **p < 0.01, ***p < 0.001.

After standardizing the main variables and controlling for gender, grade, and T1 negative emotions, a longitudinal chain mediation analysis was conducted using PROCESS Model 6. The regression results are presented in Table 2. T1 physical activity was positively associated with T2 exercise self-efficacy (*β* = 0.355, *p* < 0.001) and T3 psychological resilience (*β* = 0.210, *p* < 0.001) and negatively associated with T3 negative emotions (*β* = −0.192, *p* < 0.001). T2 exercise self-efficacy was positively associated with T3 psychological resilience, *β* = 0.225, *p* < 0.001, and negatively associated with T3 negative emotions, *β* = −0.173, *p* < 0.001. In addition, T3 psychological resilience was negatively associated with T3 negative emotions, *β* = −0.105, *p* < 0.01. These findings suggest that higher levels of T1 physical activity predicted greater T2 exercise self-efficacy and T3 psychological resilience, which in turn were associated with lower levels of T3 negative emotions. The tested model is illustrated in Figure 3.

**Table 2:** Regression results for the longitudinal chain mediation model.

Variables	T2 exercise self-efficacy		T3 psychological resilience		T3 negative emotions	
	*β*	*t*	*β*	*t*	*β*	*t*
Gender	−0.051	−0.844	−0.005	−0.082	−0.019	−0.345
Grade	−0.068	−1.665	−0.023	−0.538	−0.018	−0.480
T1 negative emotions	−0.256***	−8.404	−0.124***	−3.788	0.353***	11.959
T1 physical activity	0.355***	11.663	0.210***	6.204	−0.192***	−6.219
T2 exercise self-efficacy			0.225***	6.417	−0.173***	−5.400
T3 psychological resilience					−0.105**	−3.471
*R* ^2^	0.237		0.182		0.349	
*F*	67.830***		38.670***		77.597***	

Standardized coefficients are reported. Gender, grade, and T1 negative emotions were entered as covariates. **p < 0.01, ***p < 0.001.

The bias-corrected bootstrap method with 5,000 resamples was used to examine the significance of the total, direct, and indirect effects. An indirect effect was considered statistically significant when the 95% bootstrap confidence interval did not include zero. As shown in Table 3, the total effect of T1 physical activity on T3 negative emotions was significant, Effect = −0.284, 95% CI [−0.341, −0.228]. After including T2 exercise self-efficacy and T3 psychological resilience in the model, the direct effect remained significant (Effect = −0.192, 95% CI [−0.253, −0.132]), indicating a partial mediation effect.

**Table 3:** Total, direct, and indirect effects of T1 physical activity on T3 negative emotions.

Effect type	Effect	Boot SE	95% CI LL	95% CI UL
Total effect	−0.284	0.029	−0.341	−0.228
Direct effect	−0.192	0.031	−0.253	−0.132
Total indirect effect	−0.092	0.014	−0.121	−0.065
Indirect effect 1	−0.061	0.012	−0.087	−0.038
Indirect effect 2	−0.022	0.007	−0.037	−0.008
Indirect effect 3	−0.008	0.003	−0.015	−0.003

Values are standardized coefficients. Bootstrap confidence intervals were based on 5,000 resamples. CI, confidence interval; LL, lower limit; UL, upper limit. Indirect effects are significant when the 95% bootstrap CI does not include zero. Indirect effect 1 represents the path from T1 physical activity to T3 negative emotions through T2 exercise self-efficacy. Indirect effect 2 represents the path from T1 physical activity to T3 negative emotions through T3 psychological resilience. Indirect effect 3 represents the sequential mediating path from T1 physical activity to T2 exercise self-efficacy, then to T3 psychological resilience, and finally to T3 negative emotions.

The total indirect effect was also significant; Effect = −0.092, 95% CI [−0.121, −0.065]. Specifically, the indirect effect through T2 exercise self-efficacy was significant (Effect = −0.061, 95% CI [−0.087, −0.038]), indicating that T1 physical activity predicted lower T3 negative emotions by enhancing T2 exercise self-efficacy. The indirect effect through T3 psychological resilience was also significant (Effect = −0.022, 95% CI [−0.037, −0.008]), suggesting that T1 physical activity predicted lower T3 negative emotions by increasing T3 psychological resilience. Furthermore, the sequential indirect effect through T2 exercise self-efficacy and T3 psychological resilience was significant (Effect = −0.008, 95% CI [−0.015, −0.003]). This result indicates that T1 physical activity may first enhance T2 exercise self-efficacy, which subsequently strengthens T3 psychological resilience and ultimately reduces T3 negative emotions. Together, these findings support the hypothesized longitudinal chain mediation model.

## 4 Discussion

### 4.1 Main findings

To examine the longitudinal association between physical activity and negative emotions among college students and to clarify the mechanisms underlying this association, the present study conducted a three-wave longitudinal survey over 1 year. The findings first showed that higher levels of physical activity significantly and negatively predicted subsequent negative emotions among college students. In other words, students who reported higher levels of physical activity tended to report lower subsequent levels of depression, anxiety, and stress. Thus, Hypothesis 1 was supported. This finding is generally consistent with previous evidence suggesting that physical activity contributes to better mental health and lower symptoms of depression and anxiety (Singh et al., 2023; Rebar et al., 2015). Prior research has shown that physical activity is not only a health-promoting behavior but also an important protective resource for college students’ mental health (Herbert, 2022; Yin et al., 2025). On the one hand, regular physical activity may improve physical functioning, sleep quality, and stress-response regulation, thereby helping students alleviate tension associated with academic pressure, interpersonal adjustment, and uncertainty about the future (Xiang et al., 2024). On the other hand, physical activity often involves goal setting, behavioral persistence, positive feedback, and social interaction. These experiences may enhance positive emotional experiences and engagement in real-life activities while reducing negative rumination and emotional avoidance (Worley et al., 2023). For college students, physical activity may also serve as a daily, low-cost, and sustainable form of emotion regulation, helping them obtain more stable psychological support when coping with academic and life stress (Huang et al., 2025b; Hu et al., 2025b). Therefore, the longitudinal findings of this study further suggest that physical activity is not merely correlated with negative emotions among college students, but may also predict changes in later negative emotions over time, providing stronger longitudinal evidence for the role of physical activity in promoting college students’ mental health.

Second, this study found that exercise self-efficacy mediated the association between physical activity and negative emotions among college students, supporting Hypothesis 2. This result suggests that physical activity may be associated with lower negative emotions partly through higher exercise self-efficacy. This finding is consistent with previous studies showing that physical activity is positively related to exercise self-efficacy and that self-efficacy is closely associated with better psychological adjustment and lower emotional distress (Wei et al., 2024; Chacón et al., 2024). It also accords with social cognitive theory, which emphasizes that successful behavioral experiences are an important source of efficacy beliefs (Bandura, 2001). During physical activity, college students may gradually strengthen their beliefs in their exercise capacity and behavioral control through achieving exercise goals, improving physical fitness, receiving positive feedback, and experiencing improvements in their physical condition (Zhang et al., 2024; Ma et al., 2022).

Compared with previous studies that have mainly examined general self-efficacy or cross-sectional relationships between self-efficacy and mental health, the present study focused specifically on exercise self-efficacy and examined its role in a three-wave longitudinal framework. This distinction is important because exercise self-efficacy reflects students’ confidence in initiating and maintaining physical activity despite barriers, and may therefore represent a more proximal psychological resource linking physical activity to later emotional functioning. Higher exercise self-efficacy may not only support the maintenance of regular physical activity, but also be associated with greater initiative, perceived control, and problem-solving confidence when students face stressful situations. Conversely, students with lower exercise self-efficacy may be more likely to experience helplessness and avoidance in response to stress, which may be related to higher levels of depression, anxiety, and stress (Zhang et al., 2026b; Yuan et al., 2025). Thus, the present findings extend existing evidence by showing that exercise-related self-beliefs may help explain why students with higher levels of physical activity report lower subsequent negative emotions.

Third, this study found that psychological resilience mediated the association between physical activity and negative emotions among college students, supporting Hypothesis 3. This result is generally consistent with prior research suggesting that resilience is an important psychological resource linking physical activity to mental health outcomes. For example, previous studies have indicated that physical activity may be associated with emotional states, mental sub-health, depression, anxiety, and stress through resilience-related mechanisms. These findings collectively suggest that resilience may help individuals maintain adaptive functioning and emotional stability when facing stressful experiences (Fang et al., 2024; Gong et al., 2023). As a result, they are less likely to fall into persistent states of depression, anxiety, and stress. Physical activity itself is often characterized by challenge, goal orientation, and persistence (Xiang et al., 2026; Mastrokoukou et al., 2024). When college students participate in physical activity, they repeatedly encounter fatigue, difficulty, setbacks, and demands for self-regulation (Frederick et al., 2021). These experiences may provide a real-life context for the development of psychological resilience. Regular physical activity may help students accumulate successful experiences in coping with challenges, strengthen persistence, frustration tolerance, stress endurance, and emotional recovery, and thereby enhance psychological resilience (Hu et al., 2026e). Therefore, physical activity may not only operate through short-term emotional improvement, but may also reduce negative emotions by gradually accumulating psychological adaptive resources over time. The findings of this study further indicate that psychological resilience is an important psychological pathway linking physical activity to negative emotions among college students. They also suggest that universities may combine physical activity promotion with resilience-building programs to enhance students’ capacity to cope with stress and setbacks through sustained exercise participation.

Finally, this study found that exercise self-efficacy and psychological resilience jointly served as sequential mediators in the association between physical activity and negative emotions among college students, supporting Hypothesis 4. This finding represents one of the main contributions of the present study. A growing number of recent studies have explored similar mechanisms. For instance, some studies have examined the chain mediating roles of general self-efficacy and psychological resilience in the relationship between physical activity and academic stress among adolescents (Polizzi and Lynn, 2021), while others have investigated how physical activity may relate to resilience through self-efficacy and other psychological needs (Li et al., 2024; Zhang R., 2025). Related research has also suggested that self-efficacy, resilience, perceived stress, and coping resources may jointly explain the association between physical activity and psychological well-being (Latino et al., 2023). These studies provide important evidence that physical activity may be connected with mental health through multiple psychological resources.

Nevertheless, the present study advances this line of research by integrating exercise self-efficacy and psychological resilience into a unified longitudinal chain mediation model. Unlike studies that have focused on general self-efficacy, the present study examined exercise self-efficacy, which is more directly tied to physical activity participation. Unlike studies that have examined resilience as an isolated mediator, this study further tested whether exercise self-efficacy and psychological resilience operate sequentially. The results suggest that the association between physical activity and negative emotions may not operate through a single psychological pathway. Rather, it may reflect a progressive psychological resource-development process in which physical activity is associated with stronger exercise-related efficacy beliefs, exercise self-efficacy is linked to greater psychological resilience, and psychological resilience is further associated with lower negative emotions.

This comparison with previous research highlights the originality of the present study. While earlier studies have provided valuable evidence on physical activity, self-efficacy, resilience, and mental health, the present study extends the literature by examining these variables simultaneously in a three-wave longitudinal design among Chinese college students. By clarifying the sequential role of exercise self-efficacy and psychological resilience, this study provides a more nuanced theoretical explanation of how physical activity is linked to negative emotions and offers practical implications for campus-based mental health promotion programs that combine physical activity participation with the cultivation of exercise confidence and resilience.

### 4.2 Strengths and limitations

This study investigated the longitudinal associations between physical activity and negative emotions among college students, with particular attention to the chain mediating roles of exercise self-efficacy and psychological resilience. Several strengths should be noted. First, this study adopted a three-wave longitudinal design over 1 year, which allowed for a more rigorous examination of the temporal associations among physical activity, exercise self-efficacy, psychological resilience, and negative emotions than would be possible in a purely cross-sectional design. Second, this study focused on college students, a population undergoing important developmental transitions and facing multiple academic, interpersonal, and career-related stressors. The findings therefore provide meaningful evidence for mental health promotion in higher education settings. Third, this study contributes to the growing literature on physical activity and mental health by examining a theoretically informed serial mediation model. Although recent studies have increasingly examined psychological mechanisms linking physical activity to depressive symptoms, anxiety, stress, and psychosocial well-being, fewer studies have simultaneously considered exercise-specific self-beliefs and broader adaptive psychological resources within a longitudinal framework. By identifying exercise self-efficacy and psychological resilience as sequential mediating variables, this study provides additional evidence for how physical activity may be associated with later emotional functioning through the accumulation of psychological resources.

However, several limitations should also be acknowledged. First, although the longitudinal design provides stronger evidence than cross-sectional studies, the observational nature of this research does not allow causal conclusions. Therefore, the findings should be interpreted as longitudinal associations rather than definitive causal pathways. In particular, although physical activity was assessed at T1 and exercise self-efficacy was assessed at T2, psychological resilience and negative emotions were both assessed at T3. This measurement arrangement limits the ability to establish a clear temporal order between psychological resilience and negative emotions. Thus, the mediating role of psychological resilience should be interpreted cautiously. Future studies should use fully time-lagged designs in which physical activity, exercise self-efficacy, psychological resilience, and negative emotions are assessed at separate waves, or employ more intensive repeated-measures designs to better clarify temporal ordering.

Second, this study used PROCESS Model 6 to test the hypothesized serial mediation model. This approach was selected because the main aim was to examine a theory-driven indirect association among clearly specified variables across three measurement waves. Regression-based mediation analysis also provides a direct and interpretable estimate of specific indirect pathways. Nevertheless, this method has limitations compared with more advanced longitudinal modeling techniques. For example, structural equation modeling, cross-lagged panel models, random-intercept cross-lagged panel models, latent growth models, or longitudinal mediation models may better account for measurement error, reciprocal effects, within-person change, and stable between-person differences. Future research should use these approaches to further test the robustness of the present findings and to examine whether the associations among physical activity, exercise self-efficacy, psychological resilience, and negative emotions operate at between-person or within-person levels.

Third, although exercise self-efficacy and psychological resilience are theoretically plausible mediators, the mechanisms linking physical activity to negative emotions are likely to be more complex. Other psychological, behavioral, and biological processes may also be involved. For example, physical activity may be associated with mental health through improved sleep quality, enhanced cognitive functioning, better emotion regulation, more stable biological rhythms, reduced physiological arousal, and increased psychosocial functioning. Previous studies on physical activity and antidepressant treatment, acute intense exercise and executive functioning, and biological rhythms in depression suggest that physical activity may influence emotional functioning through multiple interacting pathways involving cognition, neurobiological regulation, and daily rhythm stability. Therefore, future studies should construct more comprehensive models that integrate exercise self-efficacy and psychological resilience with sleep, cognitive functioning, emotion regulation, biological rhythms, academic stress, and social support.

Fourth, depression, anxiety, and stress were combined into a single construct of negative emotions in the present study. This approach is statistically acceptable because these emotional symptoms are often highly correlated and can reflect a broader negative affective state. However, depression, anxiety, and stress are also distinct psychological constructs with different symptom profiles, developmental patterns, and potential responses to physical activity. For example, physical activity may be more strongly associated with depressive symptoms through behavioral activation and positive affect, whereas its association with anxiety may be more closely related to physiological regulation, perceived control, or stress reactivity. Therefore, analyzing these three dimensions separately in future research may provide a more nuanced understanding of how physical activity relates to different types of emotional distress among college students.

Fifth, the data were collected using self-report questionnaires, and the sample was recruited through convenience sampling from universities in Hunan and Jiangxi Provinces, China. These features may affect measurement accuracy and limit the representativeness of the findings. Self-report measures may be influenced by recall bias, social desirability bias, and common method variance, while convenience sampling may restrict the extent to which the findings can be generalized to broader student populations. In addition, Chinese college students may differ from students in other cultural contexts in terms of academic pressure, campus life, family expectations, physical activity opportunities, and attitudes toward mental health. Future studies should combine subjective and objective assessments, such as accelerometers, smartphone-based activity records, ecological momentary assessment, or physiological indicators, and should replicate the present model in more diverse samples across regions, educational systems, cultural backgrounds, and age groups.

Sixth, although the statistical analyses were appropriate for the research question, the interpretation of clinical or practical relevance could be further strengthened. Reporting standardized effect sizes and confidence intervals for all direct and indirect pathways would help readers better evaluate the magnitude and precision of the observed associations. Moreover, although gender, grade, and baseline negative emotions were controlled, residual confounding cannot be ruled out. Factors such as sleep habits, academic stress, perceived social support, family environment, peer relationships, diet, screen time, sedentary behavior, and other lifestyle behaviors may influence both physical activity and negative emotions. Future studies should include a broader set of covariates to reduce residual confounding and improve the explanatory power of the model.

Finally, this study mainly examined the pathway from physical activity to negative emotions through exercise self-efficacy and psychological resilience. However, the relationship between physical activity and mental health may be bidirectional. Students with higher levels of depression, anxiety, or stress may be less motivated to participate in physical activity, which may further weaken their exercise self-efficacy and psychological resilience. Future studies could adopt cross-lagged panel models, random-intercept cross-lagged panel models, or longitudinal intervention designs to examine reciprocal relationships among these variables. Despite these limitations, the present study provides longitudinal evidence that physical activity is associated with lower negative emotions among college students, and that exercise self-efficacy and psychological resilience may help explain this association. These findings offer useful implications for campus-based mental health promotion while also highlighting the need for more rigorous longitudinal and intervention-based research.

## 5 Conclusion

Based on three-wave longitudinal data collected over 1 year, this study found that higher levels of physical activity were significantly associated with lower subsequent negative emotions among college students. After controlling for gender, grade, and baseline negative emotions, exercise self-efficacy and psychological resilience both mediated the association between physical activity and negative emotions, and together formed a chain mediating pathway. These findings suggest that physical activity is linked to lower levels of depression, anxiety, and stress, partly through higher exercise self-efficacy and psychological resilience. This study provides longitudinal evidence for the psychological pathways connecting physical activity with negative emotions and offers implications for university-based mental health promotion.

## Data Availability

The original contributions presented in the study are included in the article/supplementary material, further inquiries can be directed to the corresponding author.
